# Immunohistochemical Expression of Dual-Specificity Protein Phosphatase 4 in Patients with Colorectal Adenocarcinoma

**DOI:** 10.1155/2015/283764

**Published:** 2015-01-21

**Authors:** Jongmin Sim, Kijong Yi, Hyunsung Kim, Hyein Ahn, Yumin Chung, Abdul Rehman, Se Min Jang, Kang Hong Lee, Kiseok Jang, Seung Sam Paik

**Affiliations:** ^1^Department of Pathology, College of Medicine, Hanyang University, Seoul 133-792, Republic of Korea; ^2^Department of Surgery, College of Medicine, Hanyang University, Seoul 133-792, Republic of Korea

## Abstract

The role of dual-specificity protein phosphatase 4 (DUSP4) appears to vary with the type of malignant tumors and is still controversial. The purpose of our study was to clarify the exact role of DUSP4 expression in colorectal adenocarcinoma. We constructed tissue microarrays and investigated DUSP4 expression by immunohistochemistry. DUSP4 was more frequently expressed in adenocarcinomas and lymph node/distant metastases compared to that in normal colorectal tissues and tubular adenomas (*P* < 0.001). Mean DUSP4 expression score was significantly higher in malignant tumors than in benign lesions (*P* < 0.001). DUSP4 expression was significantly correlated with older age (*P* = 0.017), male gender (*P* = 0.036), larger tumor size (*P* = 0.014), nonmucinous tumor type (*P* = 0.023), and higher T stage (*P* = 0.040). Kaplan-Meier survival curves revealed a significant effect of DUSP4 expression on both overall survival and disease-free survival in AJCC stage I (*P* = 0.008 and *P* = 0.003, resp., log-rank test) and male gender (*P* = 0.017 and *P* = 0.049, resp., log-rank test). DUSP4 protein is frequently upregulated in colorectal adenocarcinoma and may play an important role in carcinogenesis and cancer progression and may be a marker of adverse prognosis.

## 1. Introduction

Colorectal adenocarcinoma is one of the most common types of cancer and the second cause of cancer-related deaths in industrialized countries [1–3]. Despite marked advances in the understanding of carcinogenesis and improvements in diagnostic and treatment modalities, the specific therapeutic problem still persists [4, 5]. Surgery cannot always prevent the recurrence of advanced colorectal adenocarcinoma and up to 25% of colorectal adenocarcinoma patients present with liver metastasis at the time of initial diagnosis [3]. There is no appropriate targeted therapy to improve the clinical outcome of patients with colorectal adenocarcinoma [6]. The molecular prognostic markers related to a prognosis would be of great help for patients with colorectal adenocarcinoma [7].

Dual-specificity protein phosphatase 4 (DUSP4), also known as mitogen-activated protein kinase phosphatases 2 (MKP2), is a member of the dual specificity phosphatase family which inactivates target kinases through dephosphorylating phosphoserine/threonine and phosphotyrosine residues within one substrate [8, 9]. DUSP4/MKP2 is located on chromosome 8p12-p11 [10]. DUSP4 can specifically dephosphorylate the mitogen-activated protein (MAP) kinases ERK1/2, p38, and JNK [11]. These pathways drive proliferation, differentiation, apoptosis, and inflammation [12, 13]. DUSP4 expression is observed in various human cancers including breast cancer [14, 15], colorectal cancer [8, 9, 16], pancreatic cancer [17], lung cancer [18], glioma [10], and malignant melanoma [19]. However, whether DUSP4 acts as a tumor promoter or tumor suppressor is still controversial and the consensus has not been reached on the exact role of DUSP4 expression in various human cancers.

In the present study, we investigated DUSP4 expression immunohistochemically in a large series of colorectal adenocarcinoma and evaluated the association of DUSP4 expression with clinicopathological variables, as well as the impact of DUSP4 expression on survival in patients with colorectal adenocarcinoma.

## 2. Materials and Methods

### 2.1. Patients and Specimens

A consecutive series of 439 patients with colorectal adenocarcinoma was enrolled in this study. All cases were underwent operation at the Surgery of Hanyang University Hospital (Seoul, South Korea) between January 1991 and August 2001. There were 239 male and 200 female patients. The patient age ranged from 17 years to 85 years (a mean age of 57.62 years). Out of 439 cases, tumors were located in cecum (*n* = 16), ascending colon (*n* = 64), hepatic flexure (*n* = 9), transverse colon (*n* = 21), splenic flexure (*n* = 4), descending colon (*n* = 18), sigmoid colon (*n* = 95), and rectum (*n* = 212). The size of the tumor ranged from 0.3 to 15 cm (a mean size of 5.68 cm). Tumors consisted of 418 nonmucinous adenocarcinomas and 21 mucinous adenocarcinomas. Mean follow-up interval was 5.90 years. 152 (34.6%) patients died and 287 (65.4%) patients remained alive. Six cases were stage 0, 31 cases were stage I, 154 cases were stage II, 231 cases were stage III, and 17 cases were stage IV according to the American Joint Committee on Cancer (AJCC) staging system. In addition, 23 samples of normal colorectal tissue, 50 samples of tubular adenoma, 56 samples of lymph node metastasis, and 53 samples of distant metastasis were selected to evaluate the role of DUSP4 expression in carcinogenesis and tumor progression. All tissue samples were formalin-fixed and paraffin embedded. Pathologic reports, hematoxylin-eosin stained slides, and medical records were reviewed to confirm the final diagnosis and detail clinicopathologic parameters including gender, age, tumor size, tumor type, tumor location, T stage, lymph node metastasis, AJCC stage, Dukes stage, differentiation, lymphovascular invasion, and patients' survival.

### 2.2. Tissue Microarray Construction

We used a manual tissue microarrayer (Quick Ray Set, Unitama, Seoul, South Korea) for tissue microarray construction. As previously described [20], we selected areas rich in tumor cells without necrosis by light microscopy of H&E stained slides. We punched a tissue cylinder with a 2 mm diameter from a previously marked lesion of each donor block and transferred to the recipient block (Quick Ray Set, Unitama, Seoul, South Korea). Each tissue microarray was made up of 5 × 10 samples.

### 2.3. Immunohistochemical Staining

We used a polyclonal rabbit anti-DUSP4 antibody (ab72593, Abcam, Cambridge, UK) at 1 : 150 dilution. 4 *μ*m sections were cut from tissue microarray block using Leica microtome and transferred to adhesive coated slides and deparaffinized. The staining was performed using the Bond Max automated immunostainer (Vision Biosystems, San Francisco, CA, USA). Before staining, the heat-induced epitope retrieval was performed in Bond epitope retrieval solution. Endogenous peroxidase activity was blocked using 0.3% hydrogen peroxide. The primary antibody was incubated for 30 minutes and the slides were incubated with postprimary reagent for 15 minutes at room temperature. The reactions were developed using a Bond polymer refine detection kit and followed by color development with 3,3′-diaminobenzidine tetrahydrochloride as a chromogen.

### 2.4. Interpretation of Immunohistochemical Staining

DUSP4 expression was evaluated semiquantitatively by two independent pathologists (Hyunsung Kim and Seung Sam Paik) who were blinded to the patients' clinical outcome. We categorized the cytoplasmic DUSP4 expression in terms of both staining intensity and extent, as described previously [20]. Staining intensity was graded as negative (0), weak (1), moderate (2), and strong (3), and staining extent was graded as 0% (0), 1–25% (1), 26–50% (2), 51–75% (3), and 76–100% (4). The product of intensity grade and extent grade was used as the final staining score. Thus, the maximum combined score was 12 and the minimum score was 0. For the purpose of statistical analysis, a cutoff value of 3 was adopted according to the receiver operating characteristic curve. Therefore, the samples were finally classified as either negative (score 0–2) or positive (score 3–12) for DUSP4 expression.

### 2.5. Statistical Analysis

We performed statistical analysis using the SPSS software, version 19.0 (Chicago, IL, USA). Chi-square test for linear trend, Chi-square test for independence, and Mann-Whitney *U* test were used to investigate the association between DUSP4 expression and clinicopathological features including gender, age, tumor size, tumor location, tumor type, AJCC stage, Dukes stage, T category, N category, differentiation, and lymphovascular invasion. Spearman's analysis was used to obtain correlation coefficient. The Kaplan-Meier method was used to determine overall survival and disease-free survival. Univariable survival analysis was used to compare the survival rates of subgroups with the log-rank test. Multivariable survival analysis was used to determine independent prognostic factors with the Cox proportional hazards regression model. A difference of *P* < 0.05 was considered statistically significant.

## 3. Results

### 3.1. Patterns of DUSP4 Expression

**Figure 1:**
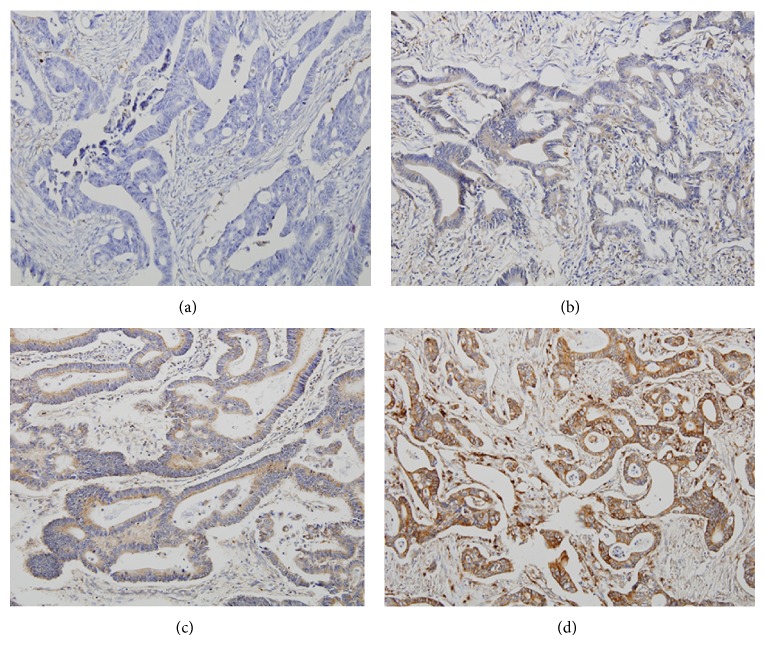
Representative microphotographs of DUSP4 immunostaining in colorectal adenocarcinoma (×200). (a) Negative, (b) weak, (c) moderate, and (d) strong. The tumor cells showed cytoplasmic DUSP4 staining.

**Figure 2:**
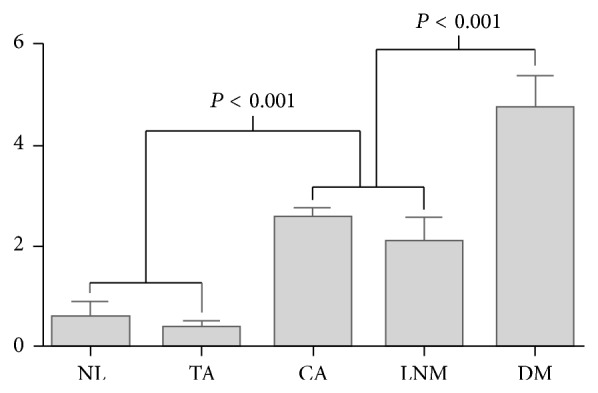
Mean DUSP4 expression score in normal colorectal tissue (NL), tubular adenoma (TA), adenocarcinoma (CA), lymph node metastasis (LNM), and distant metastasis (DM).

We evaluated DUSP4 expression in 23 samples of normal colorectal tissue, 50 samples of tubular adenoma, 439 samples of adenocarcinoma, 56 samples of lymph node metastasis, and 53 samples of distant metastasis. Various grades of cytoplasmic DUSP4 expression were observed. Representative photomicrographs of DUSP4 immunostaining in colorectal adenocarcinoma are shown in Figure 1. DUSP4 expression was positive in 2 cases (8.7%) of normal colorectal tissue and 2 cases (4.0%) of tubular adenoma; however, DUSP4 expression was positive in 166 cases (37.8%) of adenocarcinoma, 19 cases (33.9%) of lymph node metastasis, and 32 cases (60.4%) of distant metastasis (Table 1). DUSP4 was more frequently expressed in malignant tumors compared to that in benign lesions (*P* < 0.001). Mean DUSP4 expression score was 0.56 in normal colorectal tissue, 0.36 in tubular adenoma, 2.58 in adenocarcinoma, 2.10 in lymph node metastasis, and 4.75 in distant metastasis. Mean DUSP4 expression score was significantly higher in malignant tumors than in benign lesions (*P* < 0.001, Kruskal-Wallis test) (Figure 2).

### 3.2. Correlation between DUSP4 Expression and Clinicopathological Parameters

We investigated the correlation between DUSP4 expression and clinicopathological parameters to assess the clinicopathological significance of DUSP4 expression in colorectal adenocarcinoma. DUSP4 expression was significantly correlated with older age (*P* = 0.017), male gender (*P* = 0.036), larger tumor size (*P* = 0.014), nonmucinous tumor type (*P* = 0.023), and higher T stage (*P* = 0.040) (Table 2). However, there was no correlation with tumor location, N category, AJCC stage, Dukes stage, differentiation, and lymphovascular invasion.

### 3.3. Correlation between DUSP4 Expression and Overall Survival and Disease-Free Survival

**Figure 3:**
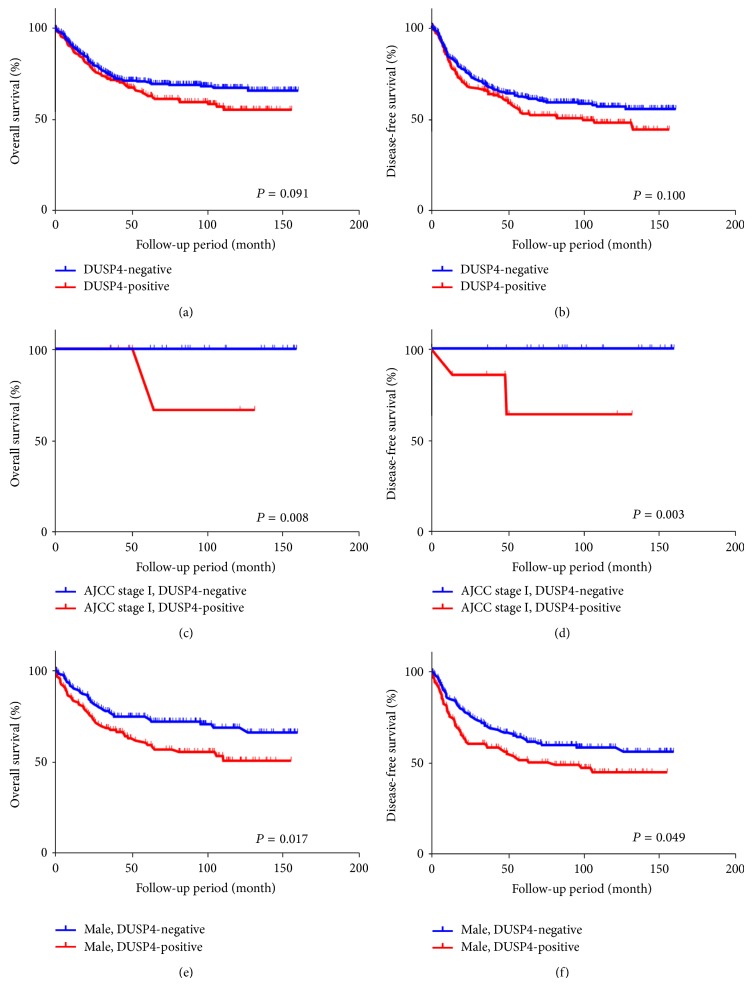
Cumulative overall and disease-free survival curves according to DUSP4 expression in all 439 patients with colorectal adenocarcinoma (a and b), AJCC stage I patients (c and d), and male gender (e and f) (Kaplan-Meier method with log-rank test).

The impact of DUSP4 expression on survival in 439 patients with colorectal adenocarcinoma was evaluated. We observed that patient age, differentiation, AJCC stage, and vascular invasion show a significant effect on overall and disease-free survival in the univariable and multivariable analyses (Table 3). There was no significant correlation between DUSP4 expression and overall survival (*P* = 0.091, log-rank test) or disease-free survival (*P* = 0.100, log-rank test) according to the Kaplan-Meier survival curves in all 439 patients with colorectal adenocarcinoma (Figures 3(a) and 3(b)). However, Kaplan-Meier survival curves revealed a significant effect of DUSP4 expression on both overall survival and disease-free survival in AJCC stage I (*P* = 0.008 and *P* = 0.003, resp., log-rank test) (Figures 3(c) and 3(d)) and male gender (*P* = 0.017 and *P* = 0.049, resp., log-rank test) (Figures 3(e) and 3(f)).

## 4. Discussion

In this study, we investigated DUSP4 expression in 23 samples of normal colorectal tissue, 50 samples of tubular adenoma, 439 samples of adenocarcinoma, 56 samples of lymph node metastasis, and 53 samples of distant metastasis and evaluated the correlation between DUSP4 expression and clinicopathological parameters and patient survival in patients with colorectal adenocarcinoma. DUSP4 was more frequently expressed in adenocarcinomas and lymph node/distant metastases compared to that in normal colorectal tissues and tubular adenomas. Mean DUSP4 expression score was significantly higher in malignant cases than in benign cases. DUSP4 expression was significantly correlated with older age, male gender, larger tumor size, nonmucinous tumor type, and higher T stage. Kaplan-Meier survival curves revealed a significant effect of DUSP4 expression on both overall survival and disease-free survival in male gender and AJCC stage I patients.

In colorectal cancer, the mitogen-activated protein kinase (MAPK) pathway is a commonly mutated pathway with 35–40% of patients having an activating mutation in* KRAS* and 5–10% of patients having an activating mutation in* BRAF* [21, 22]. Previous analysis of the gene expression profile of primary tumors revealed that* DUSP* genes are among a set of genes specific for the* BRAF* mutated tumors [8]. Dual specificity protein phosphatases (DUSPs) are a heterogeneous group of phosphatases that can dephosphorylate phosphotyrosine and phosphoserine/phosphothreonine residues within one substrate [16]. DUSPs can be divided into seven subgroups based on their sequence similarity [23]. Among them, dual specificity protein phosphatase 4 (DUSP4) is a member of the inducible nuclear MKP group and specifically dephosphorylates the mitogen-activated protein kinases ERK1/2, p38, and JNK. DUSP4 plays a crucial role in regulating the tumor-relevant MAPK pathways [9]. The exact role of DUSP4 in cancer development and progression appears to vary with the type of malignant tumors.

Recently, Saigusa et al. [9] reported that decreased expression of DUSP4 was related to metastases to liver and lung in colorectal cancer. They investigated the association between DUSP4 expression and clinical outcome in 212 patients with colorectal cancer. They described that decreased DUSP4 expression was significantly related to advanced T category, lymphatic invasion, vascular invasion, advanced stage, and distant metastasis, and increased DUSP4 expression was significantly associated with better prognosis. They concluded that DUSP4 expression was negatively correlated with factors reflecting tumor progression, including distant metastases in colorectal cancer, and suggested that DUSP4 may act as a tumor suppressor in colorectal cancer. Some reports have also described that DUSP4 may play a tumor suppressor role. Waha et al. [10] described that epigenetic downregulation of mitogen-activated protein kinase phosphatase MKP-2 relieved its growth suppressive activity in glioma cells. Chitale et al. [18] declared DUSP4 as a novel growth suppressor in* EGFR*-mutant lung adenocarcinoma. Armes et al. [24] found that DUSP4 is present in primary tumors but could be lost in early onset and high-grade breast cancers.

While several reports have revealed that DUSP4 may play a role in promoting cancer progression. Vriendt et al. [8] demonstrated that patients with high DUSP4 expression were significantly linked with a worse overall survival compared to patients with low DUSP4 expression in colorectal cancer. Gröschl et al. [16] showed that DUSP4 was frequently overexpressed in colorectal cancer with high frequent microsatellite instability (MSI-H) compared to colorectal cancer with microsatellite stable (MSS) and suggested that DUSP4 may act as an important regulator of cell growth within the MAPK pathway and may cause enhanced cell growth in MSI-H colorectal cancer. Liu et al. [14] described that overexpression of DUSP4 may play an important role in promoting the epithelial-mesenchymal transition in breast cancer and suggested that DUSP4 may be a marker of adverse prognosis.

In our study, we found that DUSP4 was more frequently expressed in cases of adenocarcinoma and lymph node metastasis compared to that in cases of normal colorectal tissue and tubular adenoma (*P* < 0.001). In addition, DUSP4 was more frequently expressed in cases of distant metastasis compared to that in cases of adenocarcinoma and lymph node metastasis (*P* < 0.001). These results suggest that DUSP4 may be involved in carcinogenesis and distant metastasis of colorectal cancer. The clinicopathological correlation analysis revealed that DUSP4 expression was significantly associated with tumor size (*P* = 0.014) and higher T stage (*P* = 0.040). These results suggest that DUSP4 may be involved in tumor progression of colorectal cancer. In survival analyses, Kaplan-Meier survival curves revealed a significant effect of DUSP4 expression on both overall survival and disease-free survival in AJCC stage I (*P* = 0.008 and *P* = 0.003, resp., log-rank test). These results suggest that DUSP4 may be a marker of adverse prognosis, especially in patients with colorectal cancer in early stage. Our results suggest that DUSP4 may play a role as a cancer promoter, not as a tumor suppressor in colorectal adenocarcinoma.

In conclusion, we investigated DUSP4 expression in a large series of colorectal adenocarcinoma. Our results were similar to the results of Vriendt et al. [8]. They demonstrated that patients with high DUSP4 expression were significantly linked with a worse overall survival compared to patients with low DUSP4 expression. However, our results showed the discrepancy with the results of Saigusa et al. [9]. They concluded that DUSP4 expression was negatively correlated with factors reflecting tumor progression and suggested that DUSP4 may act as a tumor suppressor. The exact role of DUSP4 should be further investigated in colorectal cancer and DUSP4 role as a potential novel therapeutic target for colorectal cancer should be investigated in the further study.

## Figures and Tables

**Table 1:** DUSP4 expression in NL, TA, CA, LNM, and DM (*n* = 621).

Tissue samples	*n*	DUSP4 expression			
		Negative (%) (*n* = 400)	Positive (%) (*n* = 221)	*P* value^†^	*r*_*s*_
NL	23	21 (91.3)	2 (8.7)	<0.001	0.218
TA	50	48 (96.0)	2 (4.0)		
CA	439	273 (62.2)	166 (37.8)		
LNM	56	37 (66.1)	19 (33.9)		
DM	53	21 (39.6)	32 (60.4)		

^†^Chi-square test for linear trend.

DUSP4: dual specificity protein phosphatase 4; NL: normal colorectal tissue; TA: tubular adenoma; CA: adenocarcinoma; LNM: lymph node metastasis; DM: distant metastasis; *r*_*s*_: Spearman's rank correlation coefficient.

**Table 2:** Correlation between DUSP4 expression and clinicopathological factors in colorectal adenocarcinomas (*n* = 439).

Factors	*n*	DUSP4 expression			
		Negative (%) (*n* = 273)	Positive (%) (*n* = 166)	*P* value	*r*_*s*_
Age (years)					
Mean ± SD	439	56.46 ± 13.30	59.51 ± 11.33	0.017^†^	0.114
Gender					
Male	239	138 (57.7)	101 (42.3)	0.036^‡^	−0.100
Female	200	135 (67.5)	65 (32.5)		
Tumor location					
Colon	227	137 (60.4)	90 (39.6)	0.412^‡^	−0.039
Rectum	212	136 (64.2)	76 (35.8)		
Tumor size					
Mean ± SD	439	5.45 ± 1.92	6.05 ± 2.24	0.014^†^	0.117
Tumor type					
Nonmucinous	418	255 (61.0)	163 (39.0)	0.023^‡^	−0.109
Mucinous	21	18 (85.7)	3 (14.3)		
T category					
Tis, T1, T2	46	35 (76.1)	11 (23.9)	0.040^‡^	0.098
T3, T4	393	238 (60.6)	155 (39.4)		
N category					
N0	192	123 (64.1)	69 (35.9)	0.618^*^	0.025
N1	114	68 (59.6)	46 (40.4)		
N2	133	82 (61.7)	51 (38.3)		
AJCC stage					
0, I, II	191	122 (63.9)	69 (36.1)	0.522^‡^	0.031
III, IV	248	151 (60.9)	97 (39.1)		
Dukes stage					
A, B	187	118 (63.1)	69 (36.9)	0.733^‡^	0.016
C, D	252	155 (61.5)	97 (38.5)		
Differentiation					
Well/Moderately	347	220 (63.4)	127 (36.6)	0.308^‡^	0.049
Poorly	92	53 (57.6)	39 (42.4)		
Lymphatic invasion					
Absent	186	120 (64.5)	66 (35.5)	0.388^‡^	0.041
Present	253	153 (60.5)	100 (39.5)		
Vascular invasion					
Absent	429	266 (62.0)	163 (38.0)	0.606^‡^	−0.025
Present	10	7 (70.0)	3 (30.0)		

^†^Mann-Whitney *U* test; ^‡^Chi-square test for independence; ^*^Chi-square test for linear trend.

DUSP4: dual specificity protein phosphatase 4; SD: standard deviation; AJCC: American Joint Committee on Cancer; *r*_*s*_: Spearman's rank correlation coefficient.

**Table 3:** Effect of variables on overall survival and disease-free survival in colorectal adenocarcinomas (*n* = 439).

Variables	Univariable analysis^†^		Multivariable analysis^†^	
	HR (95% CI)	P value	HR (95% CI)	P value
Overall survival				
DUSP4 expression (negative versus positive)	1.317 (0.956–1.816)	0.092	1.156 (0.833–1.604)	0.386
Patient age (<58 yrs versus ≥58 yrs)	1.856 (1.373–2.508)	<0.001	1.694 (1.216–2.360)	0.002
Differentiation (low versus high)	2.391 (1.757–3.253)	<0.001	1.678 (1.183–2.381)	0.004
AJCC stage (0, I, II versus III, IV)	3.062 (2.180–4.300)	<0.001	2.729 (1.873–3.977)	<0.001
Vascular invasion (absent versus present)	3.326 (1.561–7.090)	0.002	3.058 (1.422–6.574)	0.004
Disease-free survival				
DUSP4 expression (negative versus positive)	1.268 (0.955–1.685)	0.101	1.147 (0.859–1.533)	0.352
Patient age (<58 yrs versus ≥58 yrs)	1.491 (1.150–1.934)	0.003	1.363 (1.022–1.816)	0.035
Differentiation (low versus high)	2.115 (1.598–2.798)	<0.001	1.527 (1.113–2.095)	0.009
AJCC stage (0, I, II versus III, IV)	3.287 (2.442–4.425)	<0.001	3.122 (2.236–4.360)	<0.001
Vascular invasion (absent versus present)	2.512 (1.183–5.334)	0.016	2.280 (1.066–4.879)	0.034

^†^Cox proportional hazards model.

HR: hazard ratio; CI: confidence interval; DUSP4: dual specificity protein phosphatase 4; AJCC: American Joint Committee on Cancer.
